# Mechanistic Computational Models of MicroRNA-Mediated Signaling Networks in Human Diseases

**DOI:** 10.3390/ijms20020421

**Published:** 2019-01-19

**Authors:** Chen Zhao, Yu Zhang, Aleksander S. Popel

**Affiliations:** Department of Biomedical Engineering, Johns Hopkins University School of Medicine, Baltimore, MD 21205, USA; zhangyu@jhmi.edu (Y.Z.); apopel@jhu.edu (A.S.P.)

**Keywords:** microRNA, network motif, systems biology, mechanistic modeling, systems pharmacology, cancer, signaling pathway

## Abstract

MicroRNAs (miRs) are endogenous non-coding RNA molecules that play important roles in human health and disease by regulating gene expression and cellular processes. In recent years, with the increasing scientific knowledge and new discovery of miRs and their gene targets, as well as the plentiful experimental evidence that shows dysregulation of miRs in a wide variety of human diseases, the computational modeling approach has emerged as an effective tool to help researchers identify novel functional associations between differential miR expression and diseases, dissect the phenotypic expression patterns of miRs in gene regulatory networks, and elucidate the critical roles of miRs in the modulation of disease pathways from mechanistic and quantitative perspectives. Here we will review the recent systems biology studies that employed different kinetic modeling techniques to provide mechanistic insights relating to the regulatory function and therapeutic potential of miRs in human diseases. Some of the key computational aspects to be discussed in detail in this review include (i) models of miR-mediated network motifs in the regulation of gene expression, (ii) models of miR biogenesis and miR–target interactions, and (iii) the incorporation of such models into complex disease pathways in order to generate mechanistic, molecular- and systems-level understanding of pathophysiology. Other related bioinformatics tools such as computational platforms that predict miR-disease associations will also be discussed, and we will provide perspectives on the challenges and opportunities in the future development and translational application of data-driven systems biology models that involve miRs and their regulatory pathways in human diseases.

## 1. Introduction

MicroRNAs (miR) are a class of short (~19–24 nucleotides in length), endogenous non-coding RNA molecules with highly conserved sequences. Mature miRs are single-stranded and usually exert repressive effects on gene expression by binding primarily to the 3′ untranslated regions (UTR) of their target mRNAs through perfect or imperfect base pair complementarity [1,2]. In addition, miRs can also interact with other regions on RNAs such as 5′ UTR and coding sequences and induce non-repressive effects (e.g., translational promotion); they also interact with long non-coding RNAs and even proteins with versatile regulatory functions (reviewed in [1]). More than 1900 miRs have been identified and annotated in human to date (according to miRBase online repository), and it is estimated that about two-thirds of all protein-coding genes in human are potential targets of miRs, suggesting that miR-mediated gene regulation is universal and critical in human health and disease pathways [3,4]. Given this, dysregulation of miR expression in circulation and tissue has been observed and associated with the progression of many complex human diseases including but not limited to cancer [5,6], cardiovascular and metabolic diseases [7,8], neurodegenerative diseases [9], and infectious diseases [10]. As a result, pharmaceutical and biotechnology industries have devoted significant drug discovery efforts to pursue the therapeutic potential of miRs in various disease settings in the past few years [11].

**Figure 1:**
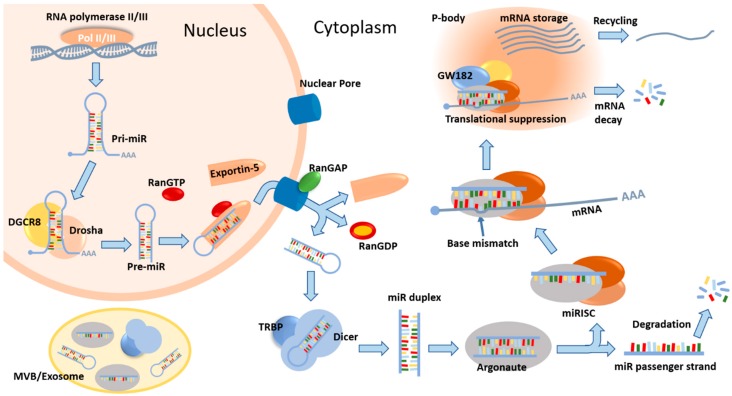
A summary of the canonical miR biogenesis pathway and miR-mediated mRNA repression. In the nucleus, genes that encode miRs are transcribed by RNA polymerases to produce primary miR transcripts (pri-miR), which are subsequently processed by the microprocessor complex (minimally consisting of Drosha and DGCR8) into precursor miRs (pre-miR). These pre-miRs are taken up by exportin-5 (RanGTP-dependent) and transported to the cytoplasm. The pre-miRs are then released and further cleaved by the endoribonuclease Dicer (assisted by the recruitment of TRBP) into double-stranded mature miR duplexes, which are later incorporated into Argonaute (AGO) proteins. The guide strand of the miR is selected and retained, whereas the passenger strand is normally degraded. Argonaute proteins (AGO1-4 in human), together with the mature single-stranded miR and several other proteins, form the miR-induced silencing complex (miRISC), within which the target mRNA binds the miR (sometimes with base mismatches). The miRISC containing the target mRNA will localize to and condense in droplet-like cytoplasmic foci called processing bodies (p-bodies) that are enriched in GW182 proteins and enzymes involved in the turnover of mRNAs. This would result in translational suppression and active degradation of the target mRNAs, and p-bodies are essential for the coordinated storage of mRNAs, as these repressed mRNAs could exit the p-bodies and re-initiate translation upon environmental stimuli. Meanwhile, some of the mature miRs, pre-miRs, mRNAs, and miR processing proteins are sorted into multi-vesicular bodies (MVB), which later become exosomes [12,18]. This figure only describes the canonical pathway of miR biogenesis. More information about non-canonical miR pathways can be found in [2]. DGCR8—DiGeorge syndrome critical region 8; RanGAP—ran GTPase activating protein; TRBP—transactivation response RNA binding protein; GW182/TNRC6A—trinucleotide repeat-containing gene 6A.

The biogenesis of miR is a tightly regulated process during which a number of proteins can potently modulate the abundance and function of miR (Figure 1) [12]. In disease settings, various underlying factors such as epigenetic alterations and gene mutations can cause abnormal miR expression, which could lead to the misregulation of a series of complex cellular pathways and thus contribute to disease progression [13]. The use of computational models, especially kinetic models (as the major focus of this review) that integrate experimental knowledge at different scales to generate systems-level understanding of the disease pathways as well as how miRs participate in these processes, can provide rich quantitative and translational insights in a cost-effective manner by considering the dynamic and multi-factorial nature of the disease [14,15]. The critical functions of miRs can sometimes be demonstrated through modeling specific network motifs which are simplified regulatory circuits that involve transcription factors (TFs), miRs and their target genes [16]. Models of this type are often based on established miR–TF–target interactions, and the steady-state model behaviors are analyzed to provide explanations to certain biological phenomena observed in experiments (e.g., bi-stability) [17]. Another broad type of computational models that study miR function focuses on the pathways that influence certain diseases. These models are generally more mechanistic, as they describe a larger number of interactions between signal transduction molecules (e.g., receptors, ligands, signaling adaptors, TFs, and miRs) that together participate in disease-related pathways and define biomarkers of disease outcome; the time-course simulations are evaluated to suggest quantitative and qualitative insights to help characterize disease mechanisms and identify optimal therapeutic targets. Both types of computational models (network motif, mechanistic) share similarities in terms of how they are constructed using systems biology techniques: (i) model components (e.g., proteins, mRNAs, and miRs) and biochemical/biophysical interactions within the model are derived from experimental data; (ii) these interactions/reactions (e.g., activation, inhibition, and binding) are represented by appropriate mathematical equations given the purpose of the model (most examples cited in this review use deterministic ordinary differential equations—ODEs); (iii) numerical values of the parameters governing the equations are identified or estimated from experimental data, literature search, or computer optimization; (iv) the models are simulated in scenarios that mimic disease conditions reflected by changes in the model inputs (e.g., initial conditions and reaction parameter values). In addition, most mechanistic models require a step of calibration during which some parameter values are further optimized by calibrating time-course model simulations (e.g., expression of miRs over time in response to stimuli) against quantitative experimental data using curve fitting algorithms. In terms of model outputs, the goal of both model types is to generate quantitative simulations that can reflect and predict experimental data, phenomenologically and mechanistically.

The application of quantitative and mechanistic systems biology models has been instrumental in modern translational research, in terms of generating and testing new hypotheses, uncovering novel mechanisms and features that are otherwise hidden in the complex cellular regulations, and expediting drug discovery at various stages such as target identification and preclinical as well as clinical development [19,20,21,22,23,24]. The same concept and methodology when applied to miR research have greatly advanced our understanding of how miRs regulate cellular phenotypes and disease pathways, as demonstrated by a series of kinetic computational models developed over the past decade for various disease contexts. Herein, we will first analyze the recent modeling examples of miR-mediated network motifs and how phenotypic cell behaviors arise from these small networks. We will then look at how different steps in miR biogenesis and mRNA repression are mechanistically implemented in quantitative models, as well as the incorporation of these details into a complex disease pathway to suggest novel disease-specific insights, especially when some of the biogenesis mechanisms are dynamically regulated by the pathway. Lastly, we will discuss potential challenges and opportunities in the field of model-driven miR translational research when multiple modeling subtypes and methodologies are integrated.

## 2. Modeling miR Function in Network Motifs

Network motifs are the repetitive regulatory network patterns that exist in large gene regulatory networks [25,26,27], and miRs play important roles in these network motifs through the interactions with their mRNA targets and transcription factors [16,28,29]. Most commonly, network motifs exist in the form of feedforward and feedback loops and contribute to a wide range of stability behaviors such as biological noise reduction (homeostasis maintenance) and multi-stability phenotypic switches; more details about miR network motifs are reviewed in [16] and [28]. Following the identification of miR–target interactions through experimental or bioinformatics approaches, kinetic models of miR network motifs can be mathematically constructed by defining and representing each molecular species, including miR, protein or TF, and mRNA, in a system of ODEs to recapitulate a simplified biological network, and the model can be simulated using either deterministic or stochastic algorithms to study its steady-state stability behaviors or dynamical response to biological noises in specific contexts [17].

**Figure 2:**
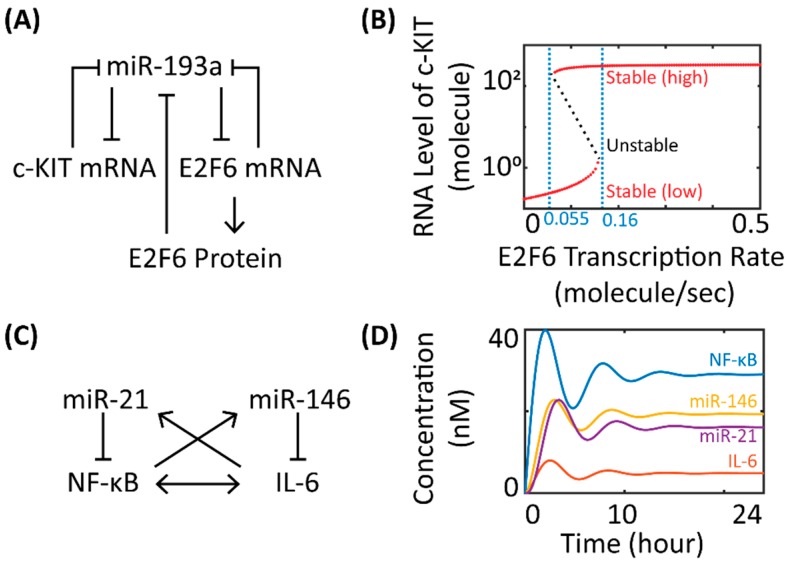
Examples of miR network motif models. (**A**) Gene regulatory network of miR-193a, c-KIT mRNA, E2F6 mRNA and protein in ovarian cancer stem cells; (**B**) a sample bifurcation diagram of the steady-state level of c-KIT mRNA with varying E2F6 transcription rates (stable and unstable steady states are shown in red and black, respectively; critical values of E2F6 transcription rate are labeled in blue; graph and results are re-created based on the model in [33]); (**C**) gene regulatory network of miR-21, miR-146, NF-κB, and IL-6 during inflammation; (**D**) simulation results of the dynamic behavior of the molecular species in the network over 24 h (graph and results are re-created based on the model in [35]). (A and C) “→” means stimulation, “––|” means inhibition.

The simulated stability behaviors of miR network motifs can often provide quantitative insights to help understand the actual phenotypic patterns observed in experiments. Kim et al. showed in a simple mathematical model that the bi-stability of a gene regulatory network controlled by miR-451 and glucose could correspond to either cell migration or proliferation in glioblastoma multiforme [30]. Lu et al. and Jolly et al. developed dynamical miR network motif models to study the stability behavior of gene regulatory networks that control epithelial-to-mesenchymal transition (EMT) [31,32]. Lu et al. demonstrated that the three stable steady states of the miR-200/ZEB and miR-34/SNAIL co-regulatory networks could be associated with the three distinct phenotypes (E—epithelial, M—mesenchymal, and E/M—hybrid) of cell fate during EMT [32]. In a follow-up study, Jolly et al. coupled the miR-200/ZEB motif with LIN28/let-7 circuit and predicted that cells in the hybrid E/M state with intermediate levels of LIN28 and let-7 were most likely correlated with high pluripotency, a key feature of cancer stem cells [31]. In another study that investigated miR in the determination of cancer cell phenotype, Cheng et al. used bifurcation analysis for a network motif model consisting of miR-193a, c-KIT, and E2F6 in ovarian cancer cells (Figure 2A) to show a bi-stable switch-like behavior of c-KIT in response to E2F6 levels, offering novel therapeutic insights for a miR-mediated network involving the ceRNA (competing endogenous RNA) mechanism in tumorigenesis [33,34]. As shown in Figure 2B, increasing (decreasing) the E2F6 transcription rate beyond the higher (lower) end of the critical values will cause the stability of the RNA level of c-KIT to move away from the low (high) expression state, skipping the intermediate unstable region between two critical values, and finally settle at the high (low) expression state. This model feature suggested that ovarian cancer cells could turn on or off the stem cell marker (c-KIT) to promote or repress cell growth in response to changes in intracellular E2F6 activation, which could be significantly regulated by cancer-specific signals such as estrogen signaling and miR-193a expression [33].

Oscillations are another common dynamical behavior produced by miR network motif models. Nandi et al. constructed two different kinetic models of circadian clock based on literature knowledge and showed that the addition of miRs can profoundly influence the amplitude and frequency of the circadian oscillators [36]. Moore et al. demonstrated in a nested miR network motif model that the oscillatory behavior of p53 induced by DNA damage in breast cancer cells can be potently downregulated by inhibiting miR-192, miR-34a, and miR-29a, while all three miRs can form positive feedback loops with p53 [37]. In addition, Xue et al. used a phenomenological four-component kinetic system to model the feedback mechanisms within NF-κB and IL-6 signaling pathways in inflammatory response [35]. In the model, expression of miR-146 and miR-21 was inducible by NF-κB activation and IL-6 signaling during inflammation, forming a coupled network of two negative feedback loops (Figure 2C). The resulting network showed damped oscillations over time (Figure 2D) and its dynamic trajectories were sensitive to the levels of miR-146 and miR-21, providing mechanistic insights into the potential involvement of cross-regulatory feedback loops in the homeostatic control of inflammatory response. This basic model platform can also be further enriched with additional signaling networks (e.g., NF-κB/IL-1 positive feedback, IL-6/SOCS negative feedback) to answer more complex research questions about inflammation [35,38,39]. More examples of kinetic computational models that describe miR network motifs with other functional properties (e.g., noise buffering, ceRNA) are discussed and reviewed by Lai et al. [17].

## 3. Mechanistic Modeling of miR Biogenesis and Target Interaction

The biogenesis and downstream target interaction of miR is a dynamic, multi-step process, which involves a number of modulatory proteins (Figure 1). Within the nucleus, miR genes are transcribed to produce long primary miR transcripts (pri-miRs), which are cleaved by microprocessor proteins and the products, precursor miRs (pre-miRs), are then exported to the cytoplasm. These pre-miRs are further processed by Dicer and Argonaute proteins to form single-stranded mature miRs, which are retained in the miRISC (miR-induced silencing complex) and can associate with target mRNAs through base pairing. As a result, translation of the bound mRNAs will be suppressed and these mRNAs may be protected and stored within processing bodies (p-bodies) in the cytoplasm or undergo cleavage and degradation (Figure 1) [12,40]. To more accurately describe the temporal dynamics of the generic miR pathway given its multi-step nature, Wang et al. proposed a mechanistic computational model that includes several key steps in the processing and function of miRs such as pri-miR cleavage, pre-miR transport, pre-miR cleavage, RISC loading of miR, and miRISC-mediated mRNA silencing [41]. The authors investigated both miRISC-induced mRNA decay and repression as alternative mechanisms, and they observed that in the repression scenario, the output mRNA and protein expression over time are much higher, while the rate of expression level changes are much slower. Since the repression scenario simulates the presence of p-bodies that sequester and store mRNAs, the model predictions suggested that miR-mediated translational repression in p-bodies could help facilitate cellular adaptations to microenvironmental changes in a rather progressive way to maintain homeostasis and avoid sudden shifts in gene expression [41,42]. Similarly, using the mechanistic modeling approach, Morozova and Zinovyev et al. first analyzed the dynamical properties of a basic linear model of miR action on protein translation and studied the effect of adding non-linear processes/reactions [43]; they then built a comprehensive kinetic model that accounts for various mechanisms of miR-mediated gene silencing, with a focus on miR–target interactions [44,45]. In the comprehensive model, the transitions between different mRNA states during translation were modeled by deterministic ODEs, and the presence of miRs can mechanistically modulate some of the transition rates, according to the nine different modes of miR-mediated inhibition (e.g., cap inhibition, elongation inhibition, ribosome drop-off, and mRNA decay; more details in [44]). By evaluating the relative changes in the steady states and relaxation times of three selected model outputs (time-course abundance of mRNA, protein, and ribosomes per translated mRNA), distinct kinetic patterns emerged which can be specifically correlated with the different modes of inhibition. Therefore, this detailed mechanistic model can help identify dominant mechanisms in a general scenario of miR-mediated gene silencing and offer insights to help design targeted experiments that can further decode the exact biophysical properties of specific miRs [44]. In the above two examples, the predictive insights derived from the time-course simulations largely hinge on the mechanistic nature of the models. In addition, these two models were formulated based on general miR function and biogenesis mechanisms, and can therefore be further refined accordingly to investigate in silico the function of a specific miR in the regulation of its target genes given certain physiological or pathological contexts.

## 4. Mechanistic Incorporation of miR-Mediated Regulatory Networks into Disease Pathways

As mentioned earlier, the kinetic function of miRs as post-transcriptional regulators of gene expression is highly dependent on the various co-factors that participate in the canonical biogenesis of miR (Figure 1) [12]. From the very beginning, the transcription of miR genes can be affected by promoters and repressors [18]. The pri-miRs can be sequestered away from the microprocessor by certain proteins in the nucleus (e.g., LIN28B) [46]. The expression and processing activity of microprocessor proteins are controlled by post-transcriptional regulations (e.g., phosphorylation) and engagement of specific RNA-binding proteins [18,47,48,49]. The function of miR transporter protein, exportin-5, can be induced by PI3K-dependent activation signals [50]. In the cytoplasm, the Dicer nuclease itself is the target of multiple miRs and its dysregulation has been associated with altered miR profiles and patient outcomes in a number of cancer types [12,51,52]. The abundance of AGO proteins, which are critical in maintaining the repressive function of miR–target interactions, can be regulated by numerous mechanisms at both the protein and mRNA levels [53,54,55]. Finally, a number of unconventional miRs are produced via non-canonical pathways that do not involve the microprocessor or Dicer [2,56]. Therefore, in human disease pathways, it is very likely that the function of miRs is dynamically affected at multiple nodes in highly nonlinear manners. Systems-level models which include mechanistic description of these transcriptional and post-transcriptional tunings can better assist researchers to uncover novel pathway features and pinpoint miR-related therapeutic targets for the disease, given that miRs allow targeted modulation of disease-related genes at the mRNA level while many of these targets are undruggable at the protein level. To better demonstrate the application of this idea, we will review the recent developments that focus on translational and mechanistic modeling in cancer and other disease settings. A summary of models to be discussed is shown in Table 1.

The development of cancer usually involves complex dysregulation in multiple cellular processes and pathways that could affect the expression and function of hundreds of biomolecules [73]. It has long been confirmed that many miRs, given their broad range of targets, possess potent tumor-promoting and/or tumor-suppressing functions in specific tumor contexts by regulating key pathways such as angiogenesis [74], tumor cell proliferation and apoptosis [75], metastatic migration [76], and immune response [77]. Mechanistic computational models of miRs in small intracellular regulatory networks in cancer have greatly advanced our understanding of the kinetic pathway patterns and time-course model behaviors, such as miR-34a in the activation of p53 [65], miR-205 in the modulation of chemoresistance [61], and miR-9/let-7 in the regulation of EGFR-mediated EMT [62]. Instead of looking at one or two miRs, Lai et al. took a systems biology approach by combining bioinformatics tools (e.g., miR target and TF prediction) with mechanistic modeling concepts and constructed a comprehensive kinetic model of a hub protein (p21) that is targeted by 15 different miRs simultaneously; the model was validated against data derived from a cancer cell line and successfully predicted p21 expression in various human tissues considering the tissue-specific expression patterns of the 15 miRs [68,69]. Awan et al. mechanistically modeled the downstream pathways of EGFR and IL-6 by integrating the molecular details of signal transduction and miR function, and used the model to generate insights on how different therapeutic modulations of the miR-17/92 cluster, both single-agent and combination therapies, can influence sorafenib resistance in hepatocellular carcinoma [63]. Using a similar pathway-centered approach, Li et al., in a series of studies, constructed a very detailed mechanistic pathway network model with more than 20 cellular signaling pathways (involving 18 miRs, a total of ~3500 molecular species and ~6000 reactions) that are broadly related to cancer development [70,71,72]. Molecular details about the key steps in miR biogenesis and target interaction (e.g., pri-miR and pre-miR cleavage, miR transport) were also incorporated so that variations in model inputs (e.g., individual genomic data from patients) would result in differential miR functions and pathway outcomes. This model was then fed with comprehensive in vitro and colorectal cancer patient-specific data from TCGA (The Cancer Genome Atlas) on miR and gene expression to quantitatively simulate personalized treatment response when therapeutics were given. The authors showed that the inclusion of individual miR expression profiles as inputs can significantly enhance the model’s predictive power, which reinforced the argument that miRs are quintessential in the modulation of complex disease pathways [70]. Moving from sub-cellular signal pathways to cell/tissue-level kinetics, multi-scale computational models have been developed to simulate miR-mediated tumor phenotypes using a hybrid of ODEs, PDEs, and agent-based modeling techniques [30,57,58]. In the case of glioblastoma, researchers have used such hybrid models to simulate tumor proliferation and migration patterns in response to perturbations in extracellular stimuli and intracellular miR-451-mediated pathways. These models were also used to identify potential drug targets and treatment strategies that can optimally eradicate the invasive glioma cells, by systematically investigating the dynamic interplay between intracellular signals, individual cell behaviors, the heterogeneous tumor microenvironment, and the phenotypic patterns of the whole tumor [30,57,58].

The idea that mechanistic systems biology models can help decode the complex pathway dynamics of miR-mediated cellular processes has also been explored in diseases other than cancer. Proctor et al. studied the effect of miRs in myogenesis during aging [59] and osteoarthritis (OA) [60], respectively, using kinetic computational models. Given the scope of each disease, the authors identified the high-relevancy miR–target pairs from literature and experimental validation; they then constructed several smaller miR-mediated pathways which were later merged into a larger reaction network where the smaller pathways were mechanistically connected to reflect pathway crosstalks based on experimental data. MicroRNA-based therapies were simulated under different stimuli conditions that mimicked the dysregulated cytokine environment in diseases to compare the time-course expression levels of myogenic (e.g., MyoD) and OA biomarkers (e.g., aggrecan and collagen 2) [59,60]. Using models of hypoxia, which is regarded as an essential feature in ischemic vascular disease and cancer [78], Zhao et al. [66,67] described in detail the complex signal transduction events during hypoxia-driven production of two key angiogenesis regulators (VEGF and TSP-1) [79]. The authors explicitly modeled the participation of several miRs in this process since they target key nodes in the model and their functions are dynamically regulated, both directly and indirectly, by low oxygen tension. By taking into account the clinical knowledge of various disease-related factors (e.g., let-7, Myc, and TGF-β) at the systems level, the models have proposed novel mechanisms to help explain the pathophysiology of abnormal angiogenesis in peripheral arterial disease and cancer [66,67]. In a more pharmaceutically related attempt, Sharma et al. combined a physiologically based pharmacokinetic model of PFOS (perfluorooctane sulfonate) exposure with a mechanistic model of miR-mediated BDNF (brain derived neurotrophic factor) production to investigate the quantitative impacts of PFOS-induced neurotoxicity [64]. This basic model can serve as a template for more advanced quantitative systems toxicology/pharmacology (QST/QSP) studies in modern pharmaceutical research and development, especially when miRs themselves are pursued as therapeutic leads or considered as important regulators of a drug’s function and toxicity.

## 5. Discussion

We have reviewed and analyzed the research efforts over the past decade to use computational kinetic modeling techniques to study the dynamic impact of miR-mediated regulation in human diseases. We have demonstrated the effectiveness and practicality of using small miR network motif models, which typically simplify to only a few components, to derive quantitative understanding and predictive features from the simulated network dynamics in a multitude of disease or physiological states. Through the examples discussed, we also showed that in the more mechanistic models that focus on miR-mediated signal transduction in disease pathways and beyond (e.g., cell and tissue level events), the higher degrees of molecular details and biochemical interactions embedded have greatly enabled researchers to explore a variety of model applications, such as generating alternative hypotheses of disease mechanisms, uncovering novel miR-based therapeutics targets, testing and comparing the efficacies of combination therapies, and stratifying responders versus non-responders for drugs given to a patient population. However, it should be noted that for kinetic miR models, having more mechanistic molecular/cellular details is not always correlated with greater model utility given the limited amount of measurable parameters and experimental data that can be used for model calibration. Future modeling studies should carefully consider their intended complexity and scope given the specific research questions and make use of sensitivity analysis and uncertainty reduction techniques when appropriate.

In cancer as well as many other diseases, the involvement of miRs is becoming more and more evident with the help of the latest computational tools, which can reliably predict miR-disease associations to expedite experimental discoveries [80,81,82]. Besides ranking-based methods that focus primarily on differential fold changes in microarrays [83], various computational platforms using data-driven approaches (based on publicly available miR-disease databases) with network-based or machine learning algorithms to find novel miR-disease associations have been recently developed and validated [82,84,85,86,87,88,89,90,91,92]. In addition to the commonly used miR target prediction tools (e.g., TargetScan) and datasets that record known miR deregulation in diseases (e.g., miR2Disease, HMDD) [93,94,95], several studies have further utilized protein–protein interaction database, disease-gene association database, TCGA and gene expression database, and the gene ontology and pathway database (e.g., KEGG) to infer new miR-disease associations as well as disease-driven changes in miR–mRNA interactions [96,97,98,99,100]. The wide range of miR-related databases and computational platforms available for the identification of candidate miRs associated with human diseases could provide rich inputs (at mRNA, miR, and protein levels) for the discovery and model-driven characterization of novel miR network motifs, as well as for future mechanistic modeling efforts that aim at the mathematical formulation of systems-level models of miRs in complex disease pathways. Still, researchers should carefully consider the variability in the quality and generation methods of the datasets from these public repositories during data collection, interpretation, and model formulation, and ensure that cross-verification from multiple sources as well as experimental validation should be performed as needed.

In summary, given the therapeutic nature of miRs as potent modulators of gene expression, plus the increasing amount of bioinformatics resources, pathway maps, computational platforms, and predictive kinetic models being implemented and made available, it makes sense to envision that quantitative multi-scale models that mechanistically investigate miRs in human diseases and treatments can be developed to substantially advance translational miR research and drug development [11,101,102]. Inspired by the examples discussed in this review, we would like to present some theoretical perspectives on the flow of how multi-scale models, especially the ones that involve miRs, can be computationally constructed to offer translational insights at sub-cellular, cell/tissue, and whole-body levels [103]. Starting from the sub-cellular level, molecular details about the disease-related signal transduction, pathway crosstalk and pathway-miR–target regulation should be included to provide a mechanistic overview of the disease (e.g., predominant pathways, biomarkers), based on information from literature and computational databases (as discussed in the previous paragraph). Then at the cell/tissue level, appropriate model assumptions (and approaches) should be implemented considering the importance of tissue heterogeneity in the scope of research. For example, agent-based models could be chosen when tissue spatial heterogeneity is of essential interest; PDEs would be appropriate to describe the spatial diffusion and gradients of drugs and secretory signals (e.g., miRs through exosomes, cytokines) that could dynamically affect the state of cells and tissues; a lumped compartmental approach can be used when the drugs, cellular signaling, and cells are assumed to be distributed uniformly within a tissue volume [103,104,105,106]. From there, the pharmacokinetic module should be added to characterize how therapeutics are transported, metabolized, and excreted in the body with mechanistic details that describe the impact of miRs, since it has been shown that miRs can influence drug metabolism by regulating CYPs (cytochrome P450) and possibly antibody recycling by regulating the neonatal Fc receptors [107,108]. Multi-scale mechanistic models that combine these features would allow selective incorporation of patient-specific omics data (e.g., genomics, proteomics, and miR profiles) and pathology data (e.g., counts and spatial patterns of different cell types from pathology slides) as inputs to generate personalized predictions and simulate inter-patient variabilities in response to different therapeutic schemes [109,110]. In the meantime, the simulated results would also provide guidance for the design of experimental validations, through which new data can be further integrated to strengthen the model’s predictive power.

## Figures and Tables

**Table 1:** Overview of recent mechanistic computational models that were developed to investigate miR-mediated pathways in human disease with a focus on the analysis of time-course kinetics.

miRs Studied	Disease or Related Pathway	Model Description	Regulation of miR Function by Other Pathway Components	Summary of Model Objectives	Ref.
miR-451	Glioma; AMPK pathway	^a^ Multi-scale model using ODE, PDE (and ABM in [57])	miR production	Simulate glioma development in response to changes in glucose and miR-451/AMPK axis	[30,57]
miR-451	Glioma; AMPK pathway	^a^ Multi-scale model using ODE, PDE and ABM	miR production	Simulate efficacies of therapies and identify optimal treatment strategies to eliminate invasive cells	[58]
miR-1, miR-181, miR-378, miR-143	Myogenesis; regulation of MyoD	^b^ Mechanistic network model using ODE *	miR production	Simulate the expression of MyoD under different combinations of miR expression	[59] Available in BioModels ^1^
miR-140	Osteoarthritis	^b^ Mechanistic network model using ODE *	miR production and degradation	Simulate the protective effect of miR-140 under various combinations of cytokine stimulation	[60] Available in BioModels ^2^
miR-205	Cancer; E2F1 pathway	^b^ Mechanistic network model using ODE	miR production	Identify pathway gene signatures that are associated with drug resistance	[61]
miR-9, let-7	Lung cancer; EGFR pathway	^b^ Mechanistic network model using ODE *	miR production	Simulate the impact of oncogenic mutations on miR expression	[62]
miR-17/92 cluster	HCC; EGFR and IL-6 pathways	^c^ Mechanistic signal pathway model using PN	None	Simulate therapies targeting the miR-17/92 cluster to combat drug resistance	[63]
miR (general)	Neurotoxicity	^d^ Mechanistic PBPK/PD Model using ODE	miR duplex cleavage	Construct a systems toxicology model that can simulate PFOS- and miR-mediated BDNF regulation	[64]
miR-34a	Cancer; p53 pathway	^b^ Mechanistic network model using ODE	miR production	Predict temporal profiles of pathway markers and study alternative mechanisms	[65]
Let-7, miR-15a	HIF-VEGF pathway	^c^ Mechanistic signal pathway model using ODE	miR production, Dicer processing and AGO loading	Simulate cellular VEGF production under hypoxia, miR control and impact of therapies	[66]
Let-7, miR-18a	TSP-1 synthesis	^c^ Mechanistic signal pathway model using ODE	miR production, pri-miR processing, Dicer processing and AGO loading	Simulate cellular TSP-1 production under TGF-β signals, hypoxia, miR control and impact of therapies	[67]
Multiple miRs	p21 expression	^b^ Mechanistic network model using ODE	None	Simulate the dynamic influence of different miR regulations on p21 expression	[68,69]
Multiple miRs	Cancer; multiple pathways	^c^ Mechanistic signal pathway model using PN	miR production, pri-miR processing, nuclear export, Dicer processing and AGO loading	Predict patient-specific response to different therapies using comprehensive gene expression data	[70,71,72]

Examples summarized in this table include models (a) that are multi-scale with both sub-cellular and cellular kinetics, (b) that combine several network motifs into larger networks, (c) that describe one or more miR-mediated cellular signal pathways in detail, and (d) that describe PK/PD profiles of a miR-mediated process. Most mechanistic models described here are simulated based on deterministic methods (* means stochastic algorithm is also used for model simulation). PDE—partial differential equation; ABM—agent based model; AMPK—AMP-activated protein kinase; MyoD—myogenic differentiation 1; E2F1—E2F transcription factor 1; EGFR—epidermal growth factor receptor; HCC—hepatocellular carcinoma; IL-6—interleukin 6; PN—Petri Net; PBPK—physiologically based pharmacokinetics; PD—pharmacodynamics; PFOS—perfluorooctane sulfonate; BDNF—brain derived neurotrophic factor; HIF—hypoxia inducible factor; VEGF—vascular endothelial growth factor; TSP-1—thrombospondin 1; TGF-β—transforming growth factor beta. ^1^ ID—MODEL1704110000-1704110004. ^2^ ID—MODEL1610100000-1610100004 and MODEL1705170000-1705170005.
